# Supplementary material for: Pitfalls in diagnosing temperature extremes
Source: Nat Commun. 2024 Mar 18;15:2087. doi: 10.1038/s41467-024-46349-x (PMC10948863; doi:10.1038/s41467-024-46349-x)
Supplement: Supplementary file 1 — Supplementary Information [file 41467_2024_46349_MOESM1_ESM.pdf]

Supplementary information to: Pitfalls in diagnosing temperature extremes

Lukas Brunner and Aiko Voigt

Department of Meteorology and Geophysics, University of Vienna, Vienna, Austria

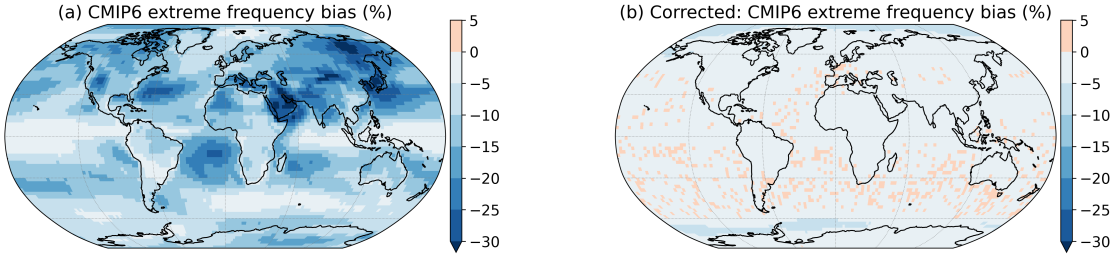

Figure S1: Bias in the frequency of temperature extremes in CMIP6. Same as top row of figure 1 in the main manuscript but for the CMIP6 multi-model mean.

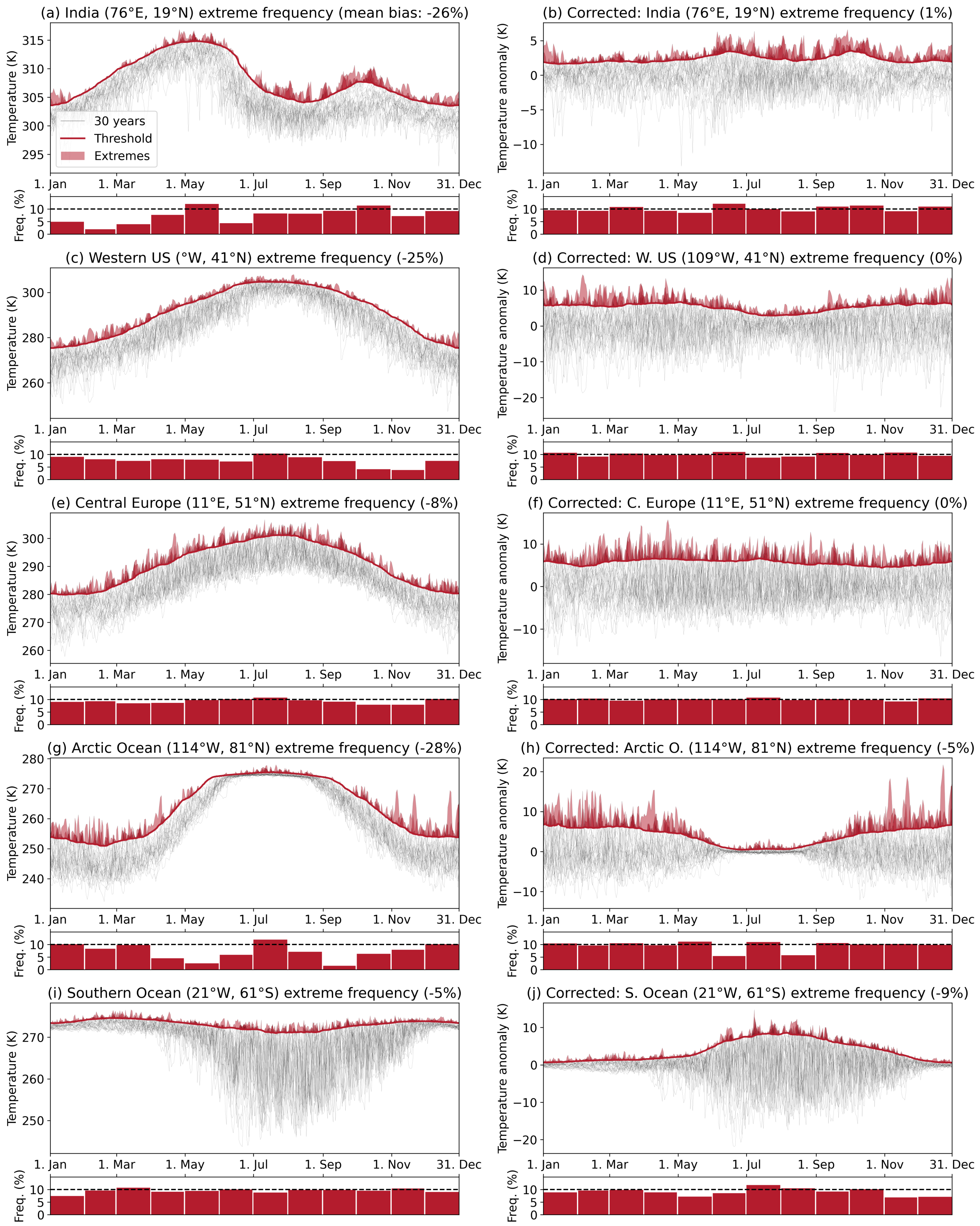

Figure S2: Bias in the frequency of temperature extremes in ERA5 for selected grid cells. Same as bottom row of figure 1 in the main manuscript but for different grid cells.

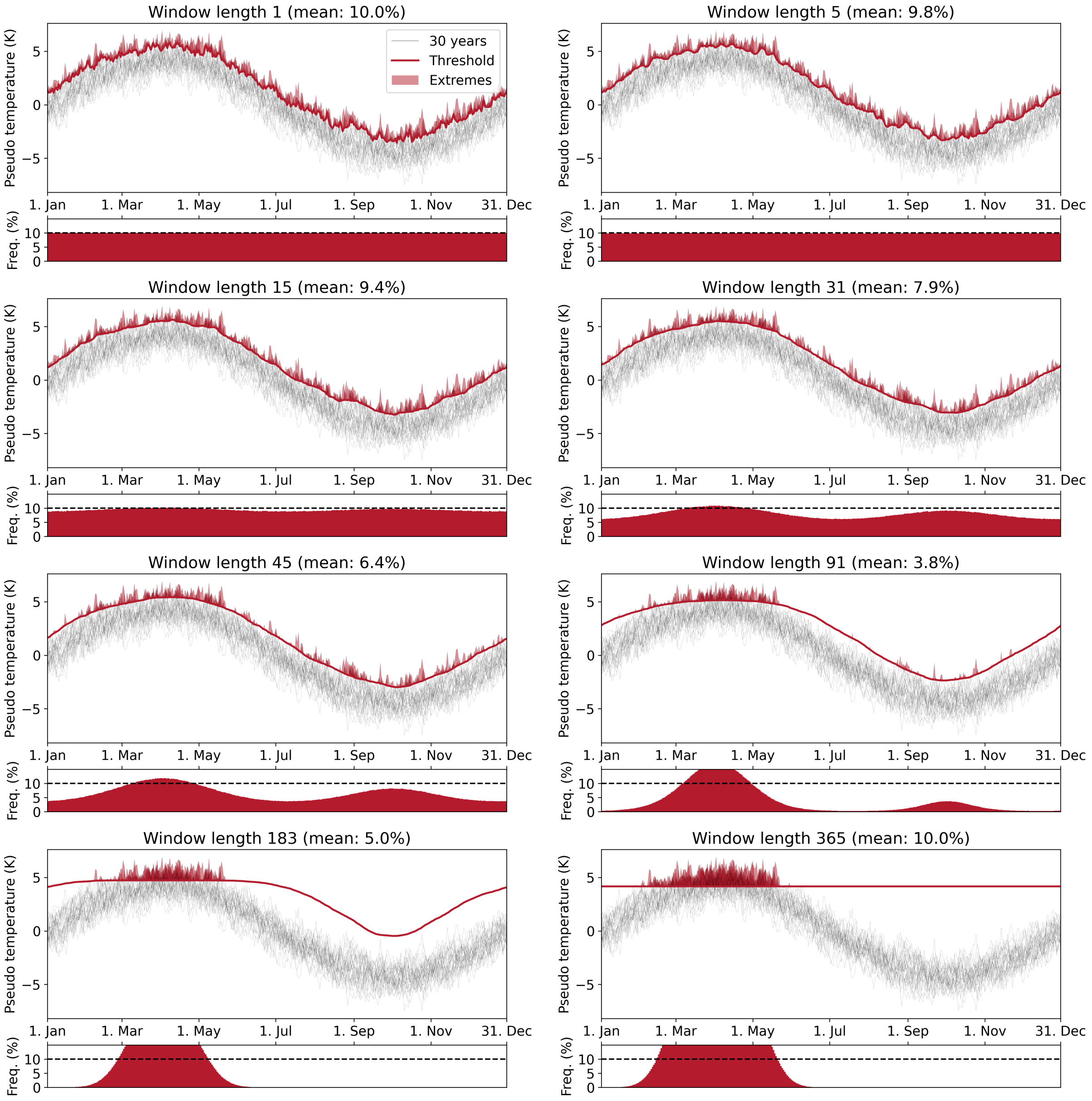

Figure S3: Threshold exceedances for different window sizes in synthetic data. Effect of different window sizes on the frequency of 90th percentile exceedances using the synthetic data with a strong seasonal cycle from figure 2 in the main manuscript. The respective top panels show threshold and exceedances for 30 seasonal cycles. The smaller bottom panels show exceedances for each day of the year averaged over all 5000 bootstrap samples.

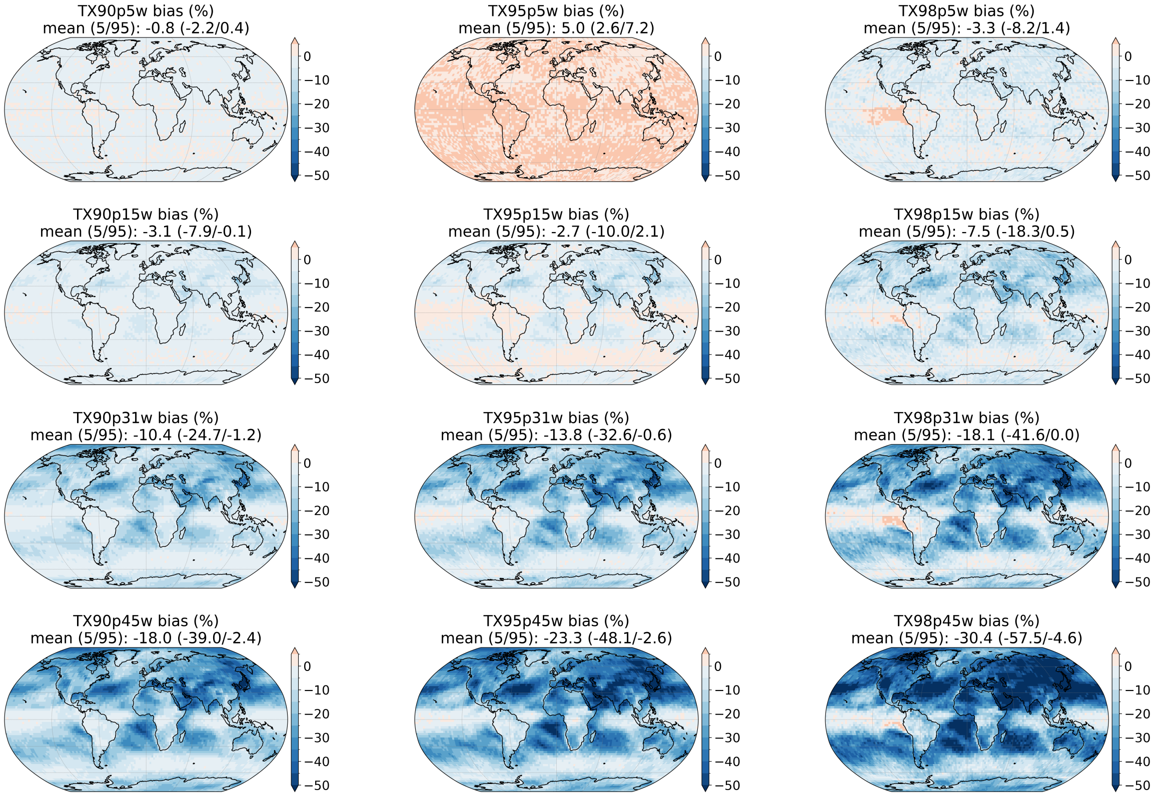

Figure S4: Bias in the frequency of temperature extremes in ERA5 for different percentiles (columns) and windows (rows). Same as figure 1a in the main manuscript but for different percentile values and window sizes as indicated. The overestimation of frequency, e.g., for TX95p5w is due to the limited number of samples (see also figure S8).

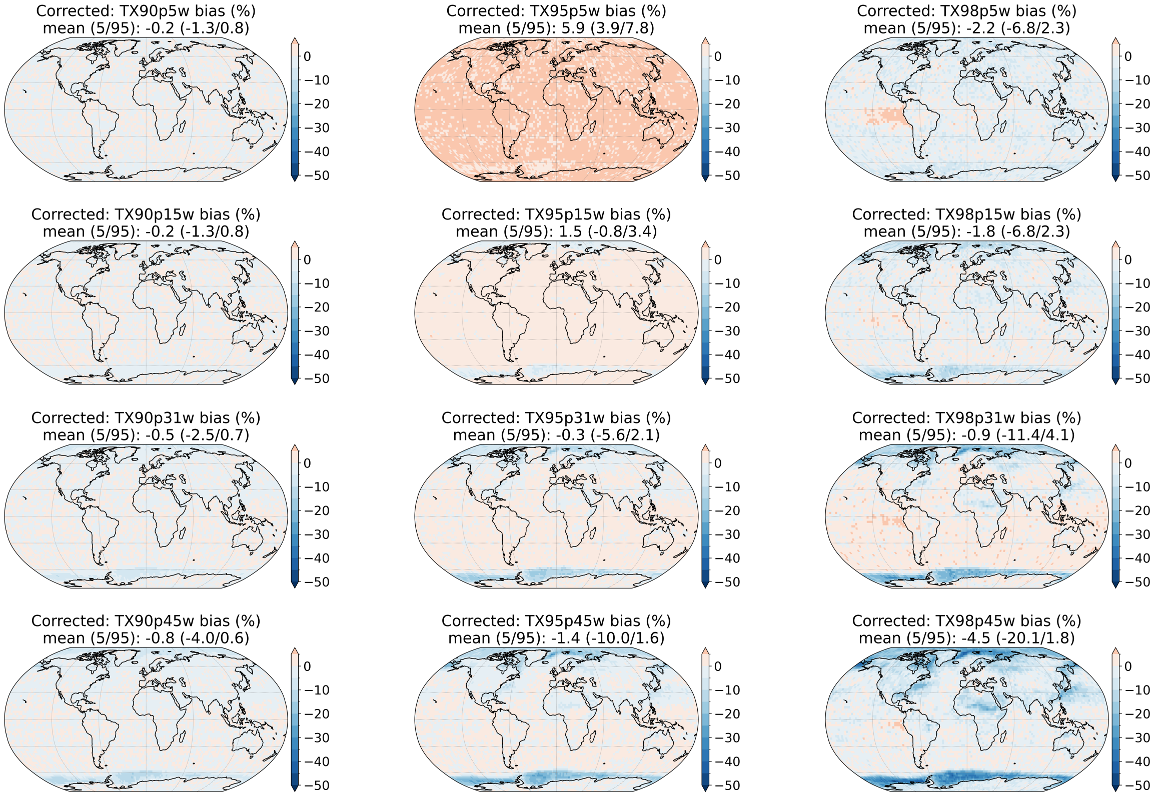

Figure S5: Corrected: Bias in the frequency of temperature extremes in ERA5 for different percentiles (columns) and windows (rows). Same as figure S4 but with the seasonal cycle removed before threshold calculation.

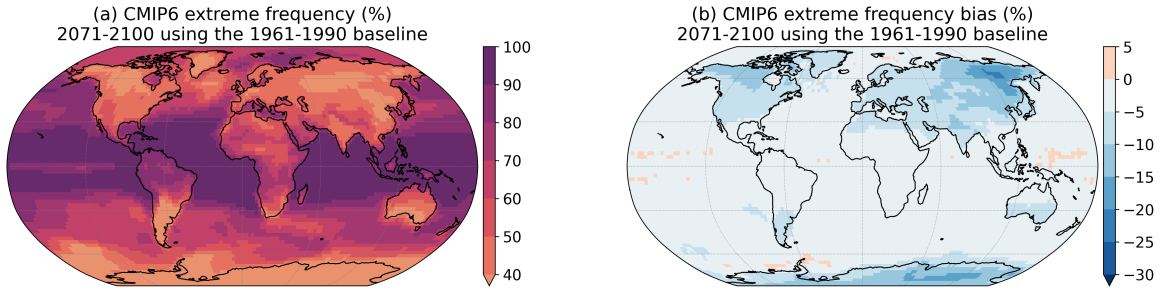

Figure S6: CMIP6 extreme temperature frequency and bias in 2071-2100 using a fixed baseline. (a) CMIP6 multi-model mean extreme frequency in 2071-2100 using 1961-1990 as baseline for calculating the threshold. (b) Bias in the extreme frequency calculated as the relative difference to the corrected case.

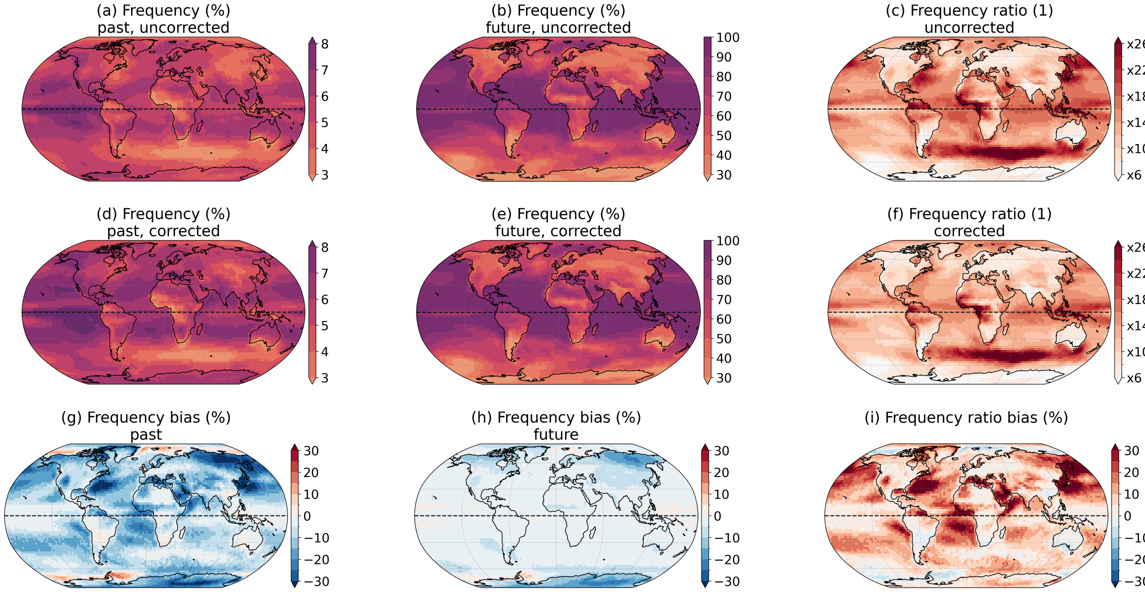

Figure S7: CMIP6 multi-model mean extended summer heatwave frequency, bias, and ratio. (a,b,d,e) CMIP6 multi-model mean frequencies for the indicated cases. Frequency bias past: g=(a-d)/d. Frequency bias future: h=(b-e)/e. Frequency ratio: c=b/a. Frequency ratio corrected: f=e/d. Bias in the frequency ratio: i=(c-f)/f. (i) is the same as figure 5d in the main manuscript but without the ocean mask.

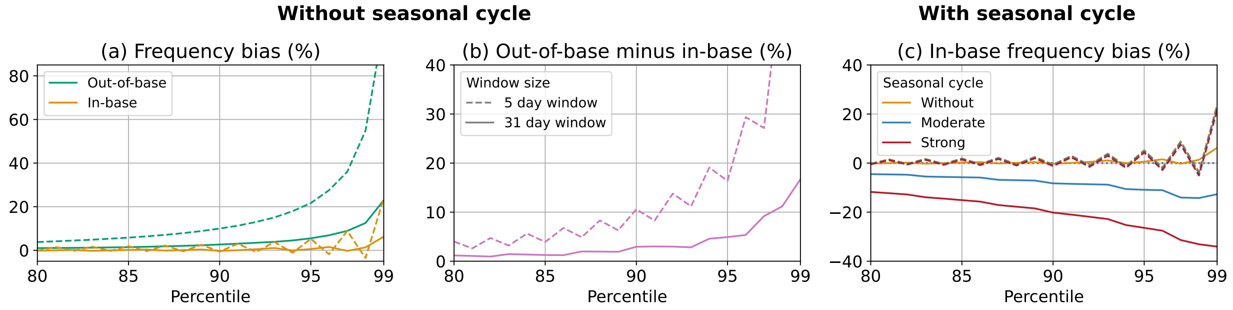

Figure S8: Bias in the exceedance frequency for different percentiles in synthetic data when using the "linear" method. Same as figure 2 in the main manuscript but using NumPy's "linear" method instead of the "weibull" method for the empiric percentile calculation (see methods for details).

Table S1: List of 26 CMIP6 models used in the study, their variant identifier, and native calendar. For models with more than one variant we use the first variant available for both time periods. Note that all gregorian-type calendars are converted to 365 day calendars by deleting the leap days.

**Model**	**Variant**	**Calendar**
ACCESS-CM2	r1i1p1f1	proleptic gregorian
ACCESS-ESM1-5	r1i1p1f1	proleptic gregorian
AWI-CM-1-1-MR	r1i1p1f1	proleptic gregorian
BCC-CSM2-MR	r1i1p1f1	365 day
CanESM5	r1i1p1f1	365 day
CMCC-ESM2	r1i1p1f1	365 day
CNRM-CM6-1	r1i1p1f2	gregorian
CNRM-ESM2-1	r1i1p1f2	gregorian
EC-Earth3-AerChem	r1i1p1f1	proleptic gregorian
EC-Earth3	r1i1p1f1	proleptic gregorian
EC-Earth3-Veg-LR	r1i1p1f1	proleptic gregorian
EC-Earth3-Veg	r1i1p1f1	proleptic gregorian
FGOALS-g3	r1i1p1f1	365 day
GFDL-ESM4	r1i1p1f1	365 day
GISS-E2-1-G	r1i1p1f2	365 day
INM-CM4-8	r1i1p1f1	365 day
INM-CM5-0	r1i1p1f1	365 day
IPSL-CM6A-LR	r1i1p1f1	gregorian
MIROC6	r1i1p1f1	gregorian
MIROC-ES2L	r1i1p1f2	gregorian
MPI-ESM1-2-HR	r1i1p1f1	proleptic gregorian
MPI-ESM1-2-LR	r1i1p1f1	proleptic gregorian
MRI-ESM2-0	r1i1p1f1	proleptic gregorian
NorESM2-LM	r1i1p1f1	365 day
NorESM2-MM	r1i1p1f1	365 day
TaiESM1	r1i1p1f1	365 day
